# Cold-Inducible RNA-Binding Protein but Not Its Antisense lncRNA Is a Direct Negative Regulator of Angiogenesis In Vitro and In Vivo via Regulation of the 14q32 angiomiRs—microRNA-329-3p and microRNA-495-3p

**DOI:** 10.3390/ijms222312678

**Published:** 2021-11-24

**Authors:** Eveline A. C. Goossens, Licheng Zhang, Margreet R. de Vries, J. Wouter Jukema, Paul H. A. Quax, A. Yaël Nossent

**Affiliations:** 1 Department of Surgery, Leiden University Medical Center, 2333 ZA Leiden, The Netherlands; eveline@via.demon.nl (E.A.C.G.); M.R.de_Vries@lumc.nl (M.R.d.V.); P.H.A.Quax@lumc.nl (P.H.A.Q.); 2 Einthoven Laboratory for Experimental Vascular Medicine, Leiden University Medical Center, 2333 ZA Leiden, The Netherlands; l.zhang.hlk@lumc.nl; 3 Department of Cardiology, Leiden University Medical Center, 2333 ZA Leiden, The Netherlands; J.W.Jukema@lumc.nl; 4 Netherlands Heart Institute, 3511 EP Utrecht, The Netherlands; 5 Department of Laboratory Medicine, Medical University of Vienna, 1090 Wien, Austria; 6 Department of Internal Medicine II, Medical University of Vienna, 1090 Wien, Austria

**Keywords:** *CIRBP*, 14q32 microRNAs, angiogenesis, peripheral arterial disease, HUVECs

## Abstract

Inhibition of the 14q32 microRNAs, miR-329-3p and miR-495-3p, improves post-ischemic neovascularization. Cold-inducible RNA-binding protein (*CIRBP*) facilitates maturation of these microRNAs. We hypothesized that *CIRBP* deficiency improves post-ischemic angiogenesis via downregulation of 14q32 microRNA expression. We investigated these regulatory mechanisms both in vitro and in vivo. We induced hindlimb ischemia in *Cirp^−/−^* and C57Bl/6-J mice, monitored blood flow recovery with laser Doppler perfusion imaging, and assessed neovascularization via immunohistochemistry. Post-ischemic angiogenesis was enhanced in *Cirp^−/−^* mice by 34.3% with no effects on arteriogenesis. In vivo at day 7, miR-329-3p and miR-495-3p expression were downregulated in *Cirp^−/−^* mice by 40.6% and 36.2%. In HUVECs, *CIRBP* expression was upregulated under hypothermia, while miR-329-3p and miR-495-3p expression remained unaffected. siRNA-mediated *CIRBP* knockdown led to the downregulation of *CIRBP*-splice-variant-1 (*CIRBP-SV1*), *CIRBP* antisense long noncoding RNA (lncRNA-*CIRBP*-AS1), and miR-495-3p with no effects on the expression of *CIRBP-SV2-4* or miR-329-3p. siRNA-mediated *CIRBP* knockdown improved HUVEC migration and tube formation. SiRNA-mediated lncRNA-*CIRBP*-AS1 knockdown had similar long-term effects. After short incubation times, however, only *CIRBP* knockdown affected angiogenesis, indicating that the effects of lncRNA-*CIRBP*-AS1 knockdown were secondary to *CIRBP-SV1* downregulation. *CIRBP* is a negative regulator of angiogenesis in vitro and in vivo and acts, at least in part, through the regulation of miR-329-3p and miR-495-3p.

## 1. Introduction

Peripheral arterial disease (PAD) is caused by occlusions of the arterial vasculature in the lower limbs, mainly the femoral artery, resulting in deprivation of blood flow and, thus, of oxygen and nutrients to the lower extremities [1]. Current treatment options include angioplasty procedures with stent placement and bypass surgery [2]. However, many patients with advanced PAD are not or no longer eligible for these therapies [3,4]. Therefore, novel therapeutic approaches are still required. In patients with PAD, endogenous neovascularization, the collective term for angiogenesis and arteriogenesis, is insufficient to completely recover blood flow to the leg [4]. Hence, enhancing neovascularization is a potential treatment option for patients with PAD. A recent study by Kübler et al. showed that mice deficient in the cold-inducible RNA-binding protein gene (*CIRBP* in humans or *Cirp* in mice) showed increased angiogenesis and decreased hypoxia-induced muscle damage in a hindlimb ischemia model, linked to M2 macrophage polarization [5,6]. *CIRBP* may act on neovascularization via other pathways too, which we investigated more closely in this study. 

*CIRBP* is regulated by differential stress factors, including ischemia [7,8] and, as its name suggests, temperature [7,9,10,11,12]. In fact, *CIRBP* was described in 1997 as the first cold shock protein that was induced at mild hypothermia [13], and this effect was conserved both in humans and mice [14]. *CIRBP* is an RNA-binding protein (RBP) that influences post-transcriptional processing of its target RNA [15], and its gene is located at chromosome 19 in humans and at chromosome 10 in mice. *CIRBP* contains an N-terminal RNA-binding domain and a C-terminal domain that has protein binding properties [13,16]. There are various splice variants of *CIRBP* that, in mice, show altered expression patterns in response to hypothermia [17]. The four main splice variants in humans, *CIRBP*-SV1, *CIRBP*-SV2, *CIRBP*-SV3, and *CIRBP*-SV4, have their RNA-binding domain in common but have different C-termini. However, the role of these splice variants, especially the link with microRNAs and neovascularization, needs further investigation. Furthermore, the antisense strand of the human *CIRBP* gene encodes an antisense long non-coding RNA (lncRNA-*CIRBP*-AS1). Antisense long noncoding RNAs (lncRNAs) can have several functions. Antisense lncRNAs have been shown to affect transcription and support function of their respective coding sense-strand [18]. For example, lncRNA MALAT1 has an antisense transcript TALAM1, and together they function as a sense–antisense pair [19,20]. Therefore, it is possible that either sense and antisense strands are co-transcribed and counteract or cooperate in their actions [18] or that one strand affects expression of the other strand. Whether *CIRBP* and lncRNA-*CIRBP*-AS1 have a similar “partnership” remains to be determined.

*CIRBP*, as an RBP, not only affects processing of messenger RNAs (mRNAs) but also has the ability to act in microRNA processing. Previously, our group showed that a large microRNA cluster, located on chromosome 14 (14q32 locus), plays a regulatory role in different types of vascular remodeling including atherosclerosis and restenosis but also in post-ischemic neovascularization [21,22,23,24]. This cluster is also known as the DLK1-DIO3 cluster and is conserved in mice where it is located at the 12F1 locus. *CIRBP* was shown to directly bind two precursors of 14q32 microRNAs, namely, precursor-microRNA (pre-miR)-329 and pre-miR-495 [25], thereby inducing processing into the mature microRNAs miR-329-3p and miR-495-3p. In previous studies, it was found that inhibition of the 14q32 microRNAs, miR-329-3p and miR-495-3p, increased post-ischemic neovascularization [21] and angiogenesis, in particular. At the same time, inhibition of these microRNAs also reduced post-interventional restenosis [22], potentially offering a double advantage for patients with severe PAD. Importantly, inhibition of miR-495-3p affected macrophage influx into the lesions. Therefore, the hypothesis was that inhibition of *CIRBP* leads to a decrease in mature miR-329-3p and miR-495-3p expression and, consequently, promotes post-ischemic neovascularization. 

In this study, firstly, the effect of *CIRBP* deficiency on neovascularization in vivo was investigated using a murine hindlimb ischemia model, and the effects on arteriogenesis and angiogenesis were determined, showing that mainly angiogenesis was affected. Next, human umbilical vein endothelial cells (HUVECs) were used to examine the effects of modulating the expression of *CIRBP*, its splice variants, its antisense lncRNA-*CIRBP*-AS1, and its downstream target microRNAs, miR-329-3p and miR-495-3p, and to demonstrate the effects of *CIRBP* and lncRNA-*CIRBP*-AS1 knockdown on in vitro angiogenesis. 

## 2. Results

### 2.1. Blood Flow Recovery and Neovascularization after Hindlimb Ischemia (HLI) Surgery in Cirp^−/−^ Mice

**Figure 1:**
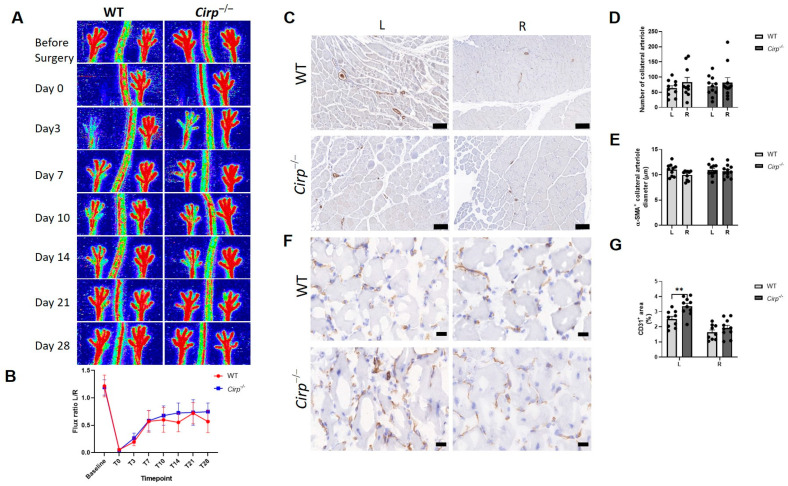
Hindlimb Ischemia (HLI) in mice. (**A**) Representative laser Doppler perfusion imaging (LDPI) of paws of wild-type (WT) mice (*n* = 10) and *Cirp* knockout (*Cirp^−/−^*) mice (*n* = 11) subjected to HLI over time. (**B**) Quantification of LDPI measurements over time, calculated as the ratio of the left (ischemic) over the right (non-ischemic) paw. (**C**) Representative images of α-smooth muscle actin positive (α-SMA^+^) arterioles in adductor muscle of mice, scale bar = 100 μm. (**D**,**E**) Quantification of the number and average diameter of α-SMA^+^ arterioles in adductor muscles. (**F**) Representative images of CD31^+^ capillaries in the soleus muscle, scale bar = 20 μm. (**G**) Quantification of the CD31^+^ area in the soleus muscle. Data are presented as the mean ± SEM; ** *p* < 0.01 by independent sample Student’s *t*-tests.

To investigate the role of *CIRBP* in neovascularization in vivo, an HLI model was induced in *Cirp* knockout (*Cirp^−/−^*) and wild-type (WT) C57BL/6 mice, and blood flow recovery was evaluated over time. No differences were observed in blood flow recovery between WT mice and *Cirp^−/−^* mice over 28 days (Figure 1A,B). 

To visualize arteriogenesis, immunohistochemical staining for α-smooth muscle actin (α-SMA) in the adductor muscle was performed (Figure 1C), and the number and diameter of α-SMA positive arterioles were quantified. Collateral density and the size of α-SMA positive arterioles of the ligated paws were similar between WT mice and *Cirp^−/−^* mice (Figure 1D,E), which is in line with the results presented in Figure 1A.

To monitor the effects of *Cirp* deficiency on angiogenesis, capillary formation was evaluated in the soleus muscles at 28 days after induction of ischemia as visualized by CD31 staining (Figure 1F). The CD31^+^ area represents the density of capillaries in the ischemic muscle. Compared to the unligated (right) paw, there was more capillary formation in the ligated (left) paw as shown in both WT mice (65.2% increase, *p* = 0.009) and *Cirp^−/−^* mice (100.5% increase, *p* = 0.003). Moreover, the CD31 positive area in the ligated paw of *Cirp^−/−^* mice was significantly higher than in WT mice (34.3% increase, *p* = 0.004), while there was no difference in the unligated paw between the two groups (Figure 1G). 

### 2.2. Ex Vivo Angiogenic Sprouting in Aorta Rings

**Figure 2:**
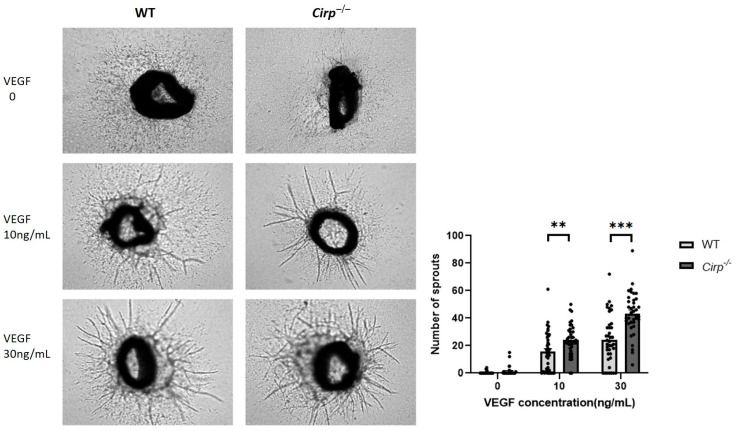
Ex vivo angiogenesis: representative images and quantification of neovessel sprouts from 7 day collagen-embedded aorta rings (40 rings per condition from 3 WT and 3 *Cirp^−/−^* mice), treated without or with vascular endothelial growth factor (VEGF) at 10 and 30 ng/mL. Data are presented as the mean ± SEM; ** *p* < 0.01; *** *p* < 0.001 by independent sample Student’s *t*-tests.

To study the effects of *Cirp* deficiency on angiogenesis ex vivo, aorta ring assays were performed. Two different vascular endothelial growth factor (VEGF) concentrations (i.e., 10 and 30 ng/mL) were used to induce sprouting in either wild-type or *Cirp* deficient aorta rings. Aorta rings from *Cirp^−/−^* mice developed more sprouts compared to the WT control rings, both in rings incubated with 10 (55.4% increase in sprouts) or 30 ng/mL VEGF (79.6% increase in sprouts) (Figure 2). Quantification of the sprouting demonstrated that for both VEGF concentrations, this difference was statistically significant (10 ng/mL VEGF, *p* = 0.004; 30 ng/mL VEGF, *p* < 0.001). 

### 2.3. MicroRNA Expression in Cirp^−/−^ Mice

**Figure 3:**
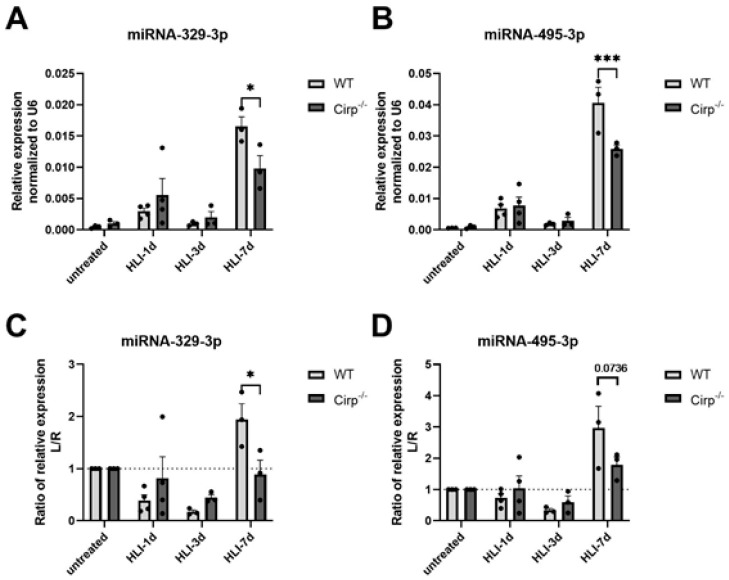
Expression of miRNA-329 and miRNA-495 in soleus muscles. (**A**,**B**) 1, 3, and 7 days after HLI surgery, mature miR-329-3p and -495-3p expression in soleus muscles of the ligated paws from WT mice (*n* = 3 for the untreated group, *n* = 4 for the HLI-1d group, *n* = 3 for the HLI-3d group, and *n* = 3 for the HLI-7d group) and *Cirp^−/−^* mice (*n* = 3 for the untreated group, *n* = 4 for the HLI-1d group, *n* = 3 for the HLI-3d group, and *n* = 3 for the HLI-7d group). Expression was normalized to U6. (**C**,**D**) The relative expression of mature miRNA-329 and -495 from mice after HLI surgery at different timepoints, calculated as the ratio of the left (ischemic) over the right (non-ischemic) paw. Data are presented as the mean ± SEM; * *p* < 0.05; *** *p* < 0.001 by two-way ANOVA.

MiR-329-3p and miR-495-3p are both highly associated with angiogenesis and regulated by *CIRBP* as has been shown previously [21,25]. To study if and how miR-329-3p and -495-3p expression changes in *Cirp^−/−^* mice after induction of hindlimb ischemia, the HLI surgery was repeated, and the mice were sacrificed at 1 day, 3 days, and 7 days after surgery, followed by RNA isolation from the soleus muscle from both the left and right paws. These early timepoints were chosen as ischemia had not resolved yet and angiogenesis was still ongoing. Although the expression of miR-329-3p and miR-495-3p increased in the ischemic soleus muscles of both mouse strains at 7 days, their expression increased significantly less in the *Cirp^−/−^* mice (40.6% less, *p* = 0.028 for miR-329-3p; 36.2% less, *p* < 0.001 for miR-495-3p; Figure 3A,B). This reduced upregulation became even more clear when looking at the ratio of expression in the ligated over the non-ligated paws, where the ratio only increased in the WT mice and not in the *Cirp^−/−^* mice (*p* = 0.027 for miR-329-3p; *p* = 0.0736 (trend) for miR-495-3p; Figure 3C,D). These differences were most evident at day 7.

### 2.4. Total CIRBP, CIRBP Splice Variants, and lncRNA-CIRBP-AS1 Expression in Hypothermia

**Figure 4:**
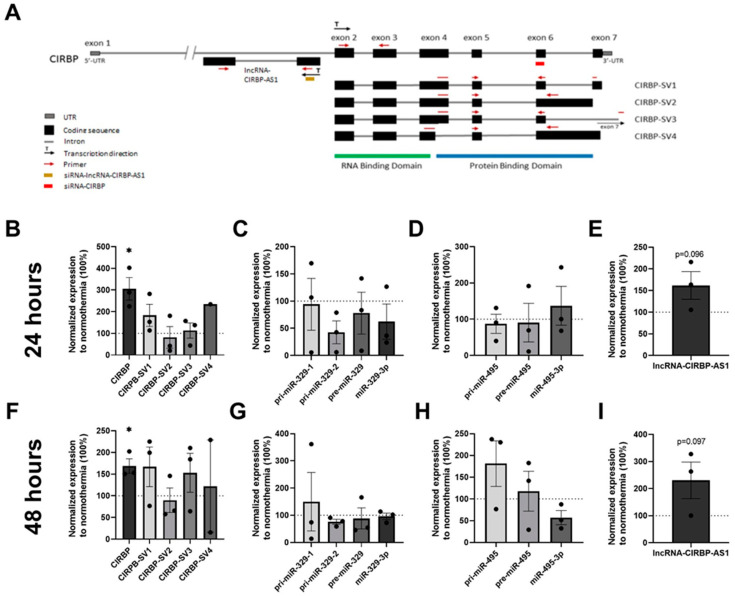
Cold-inducible RNA-binding protein gene (*CIRBP*) expression under hypothermia for 24 and 48 h: (**A**) Schematic representation of the *CIRBP* gene, its splice variants, and lncRNA-*CIRBP*-AS1 with the binding sites of primers and small interfering RNA (siRNA) indicated. Human umbilical vein endothelial cells (HUVECs) were cultured under hypothermic conditions (32 °C) for 24 and 48 h, and (**B**,**F**) *CIRBP* and *CIRBP* splice variant (*CIRBP-SV*) expression levels were measured and normalized to GAPDH. *CIRBP*-SV4 expression was below the detection limit in several experiments; (**C**,**D**,**G**,**H**) primary microRNA (pri-miR), precursor microRNA (pre-miR), and mature microRNA expression levels of miR-329 and miR-495 were measured and normalized to U6; (**E**,**I**) *CIRBP* gene antisense long non-coding RNA (lncRNA-*CIRBP*-AS1) expression level was measured and normalized to GAPDH. Data are shown as the relative expression compared to the normothermic group, presented as the mean ± SEM; * *p* < 0.05 by one-sample *t*-tests (one-tail).

The gene structure of the human *CIRBP* is shown in Figure 4A. Previous studies showed that *CIRBP* expression increased under cellular stress conditions including mild hypothermia [9]. To demonstrate that hypothermia upregulates *CIRBP*-targeted miRNAs and also the different *CIRBP* splice variants and antisense lncRNAs in HUVECs, HUVECs were subjected to mild hypothermia (32 °C) either for 24 or 48 h. Total *CIRBP* expression increased after both 24 and 48 h compared to the normothermic condition (37 °C) by 205.5% (*p* = 0.029; Figure 4B) and 68.7% (*p* = 0.028; Figure 4F), respectively. *CIRBP-SV1* expression increased by 83.1% at 24 h (*p* = 0.12) and by 67.1% at 48 h (*p* = 0.14) under hypothermia, although this upregulation was not statistically significant. However, the remaining three splice variants were not altered consistently over time under hypothermia, neither were significant changes in microRNA expression observed over time (Figure 4C,D,G,H), indicating that upregulation of *CIRBP* did not lead to additional microRNA processing.

lncRNA-*CIRBP*-AS1 did show a trend towards a 61.7% increase in expression under hypothermia after 24 h (*p* = 0.096; Figure 4E) and was even further upregulated by 130.4% after 48 h (*p* = 0.097) compared to normothermia (Figure 4I).

### 2.5. Angiogenesis Assays and RNA Expression after CIRBP Knockdown

**Figure 5:**
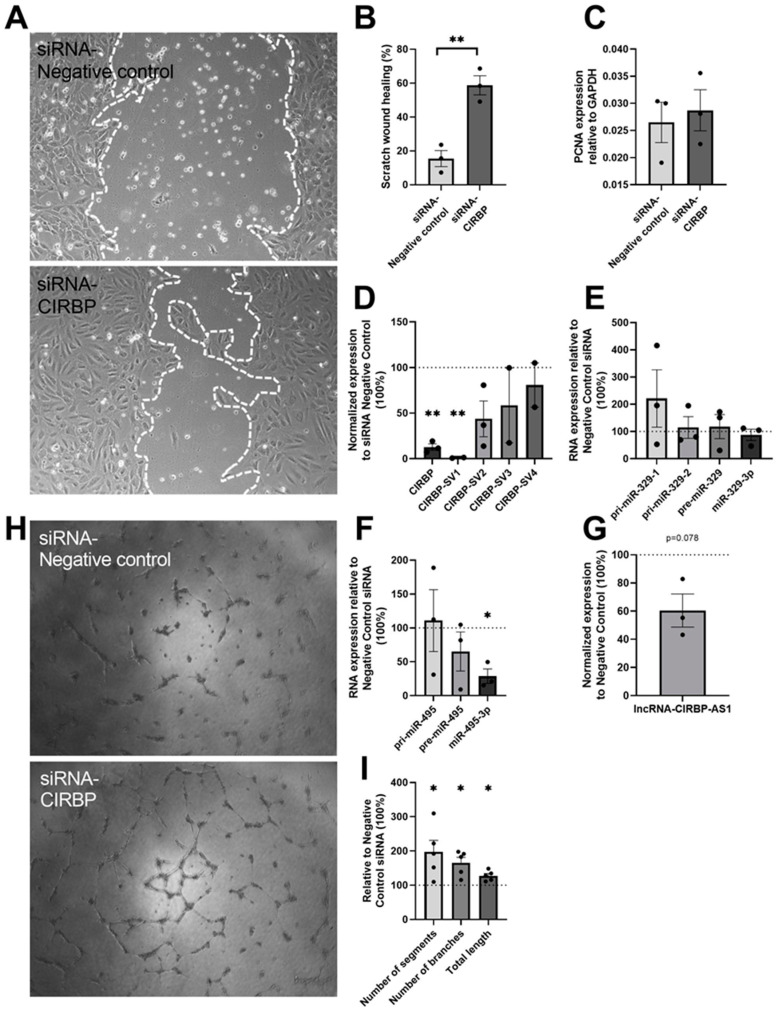
siRNA-*CIRBP* transfection for 24 h in HUVECs: (**A**) Representative images of wound healing (*n* = 3) and (**B**) quantification of the migration area treated with siRNA targeted to *CIRBP* and negative control for 24 h. White, dotted lines mark the edge of the HUVECs’ monolayer. (**C**) Proliferating cell nuclear antigen gene (*PCNA*) expression level in HUVECs as an indicator of cell proliferation. (**D**) Expression of total *CIRBP* and its splice variants after scratch assay, normalized to GAPDH. *CIRBP*-SV3 and *CIRBP*-SV4 expression were below the detection limit in one out of three experiments. (**E**–**G**) pri-miRs, pre-miRs, and mature microRNA expression levels of miR-329 and miR-495 were measured after scratch assay and normalized to U6; lncRNA-*CIRBP*-AS1 expression levels were measured and normalized to GAPDH. Data show the percentage compared to the siRNA negative control group. (**H**) Representative image of the tube-formation assay (*n* = 5) on HUVECs treated over 24 h with siRNA targeted to *CIRBP* and negative control. (**I**) Quantification of the number of segments, number of branches, and total length of the tubes of the HUVECs after siRNA treatment compared to the negative-control siRNA treatment. Data are presented as the mean ± SEM; * *p* < 0.05; ** *p* < 0.01; independent sample Student’s *t*-tests (**B**,**C**) or one-sample t-tests (two-tail) (**D**–**G**,**I**).

To investigate the role of *CIRBP* in vitro, HUVECs were treated with small interfering RNA (siRNA) targeted to *CIRBP*, and the angiogenic potential was assessed using both scratch-wound healing assays and tube-formation assays. Scratch-wound healing showed an increased (2.8-fold, *p* = 0.004) angiogenic potential after siRNA--*CIRBP* transfection (Figure 5A,B). To exclude any effects of potential *CIRBP* knockdown-induced cell proliferation, the expression of proliferating cell nuclear antigen (*PCNA*) mRNA was assessed and, indeed, no differences were observed compared to the negative control siRNA (Figure 5C). In addition, tube-formation assays also showed improvements in the number of segments (97.2%, *p* = 0.046), branches (65.4%, *p* = 0.016), and total length (27.7%, *p* = 0.014) after *CIRBP* knockdown (Figure 5H,I).

After the scratch assays, cells were collected for RNA isolation to assess the expression of total *CIRBP*, *CIRBP-SVs*, and lncRNA-*CIRBP*-AS1 as well as the two microRNAs and their precursors. As shown in Figure 4A, the siRNA-*CIRBP* was designed to target all splice variants. Total *CIRBP* levels were indeed knocked down by 87.3% (*p* = 0.002, Figure 5D). However, when looking at the individual splice variants, only *CIRBP*-SV1 was knocked down (99.0% decrease, *p* = 0.002), whereas the other splice variants remained unaffected (Figure 5D). Furthermore, a trend towards decreased lncRNA-*CIRBP*-AS1 expression (39.5% decrease, *p* = 0.078) was also observed when *CIRBP* was knocked down (Figure 5G). Although upregulation of *CIRBP* expression through hypothermia did not increase microRNA expression, a significant decrease (71.2%; *p* = 0.02; Figure 5F) was observed in the expression of miR-495-3p under *CIRBP* knockdown. Surprisingly, miR-329-3p was not significantly downregulated in response to *CIRBP* knockdown, as it was in Cirp-deficient mice. We did observe a trend towards accumulation of the primary microRNA, pri-miR-329-1, which is in correspondence with *CIRBP*’s effects on microRNA biogenesis [25].

### 2.6. CIRBP and miRNA Expression in lncRNA-*CIRBP*-AS1 Knockdown 

**Figure 6:**
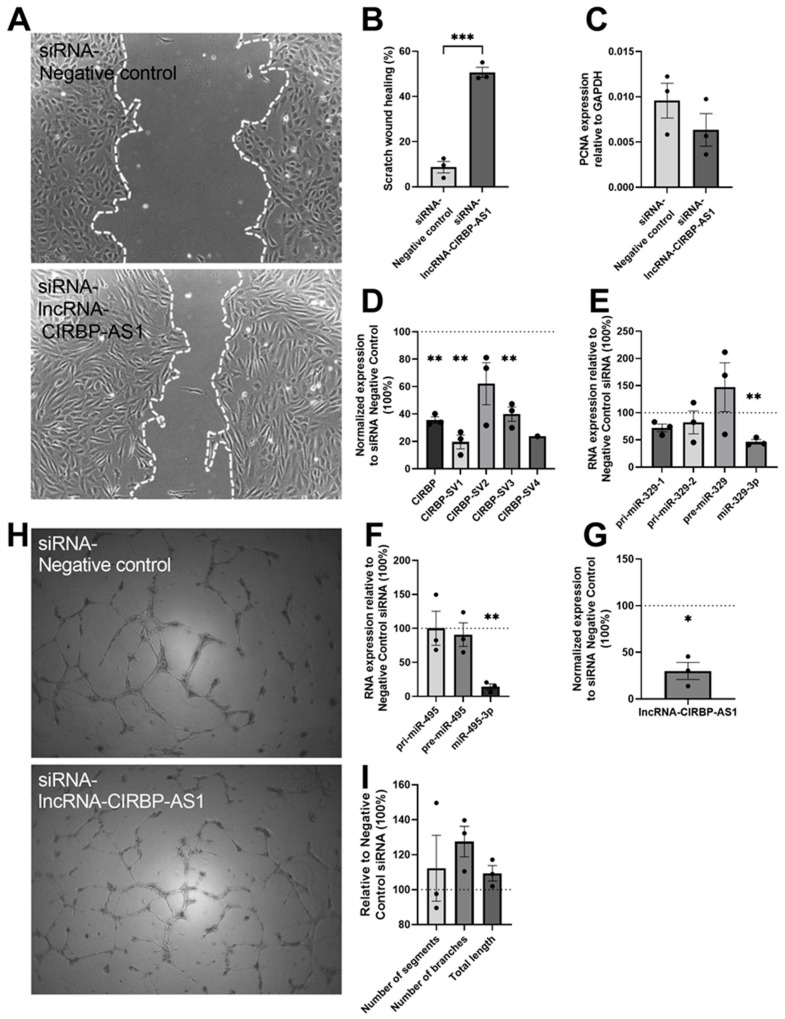
siRNA-lncRNA-*CIRBP*-AS1 transfection over 24 h in HUVECs: (**A**) Representative images of wound healing (*n* = 3) and (**B**) quantification of the migration area, treated with siRNA targeted to lncRNA-*CIRBP*-AS1 and the negative control for 24 h. White, dotted lines mark the edge of the HUVECs’ monolayer. (**C**) *PCNA* expression level in HUVECs as an indicator of cell proliferation. (**D**) Total *CIRBP* expression and its splice variant expression after the scratch assay, normalized to GAPDH. *CIRBP*-SV4 expression was below the detection limit in two out of three experiments. (**E**–**G**) After the scratch assay, pri-miRs, pre-miRs, and mature microRNA expression levels of miR-329 and miR-495 were measured and normalized to U6; lncRNA-*CIRBP*-AS1 expression levels were measured and normalized to GAPDH. Data show the percentage compared to the siRNA negative control group. (**H**) Representative image of the tube formation assay (*n* = 3) on HUVECs treated over 24 h with siRNA targeted to *CIRBP* and the negative control. (**I**) Quantification of the number of segments, number of branches, and total length of the tubes of the HUVECs after siRNA treatment compared to the negative-control siRNA treatment. Data are presented as the mean ± SEM; * *p* < 0.05; ** *p* < 0.01; *** *p* < 0.001; independent sample Student’s *t*-tests (**B**,**C**) or one-sample *t*-tests (two-tail) (**D**–**G**,**I**).

In order to determine the potential effects of lncRNA-*CIRBP*-AS1 on both angiogenesis and *CIRBP* and microRNA expression, the angiogenesis assays described above were repeated using an siRNA against the lncRNA itself. Knockdown of lncRNA-*CIRBP*-AS1 resulted in a 4.8-fold improvement in HUVEC migration (*p* < 0.001; Figure 6A,B), while no significant difference was observed in the tube formation (Figure 6H,I). The potential effect of cell proliferation on scratch-wound healing was excluded, as there were no differences in the expression of PCNA mRNA between the groups (Figure 6C). Along with the antisense lncRNA (70.0% downregulation; *p* = 0.017; Figure 6G), total *CIRBP* expression was also downregulated by 64.4% (*p* = 0.001; Figure 6D). Both *CIRBP*-*SV1* (80.4% downregulation; *p* = 0.004) and *CIRBP-SV3* (60.0% downregulation; *p* = 0.007) expression decreased, while *CIRBP-SV2* and *-SV4* were unaffected (Figure 6D). Furthermore, knockdown of lncRNA-*CIRBP*-AS1 also resulted in a 53.5% downregulation of mature miR-329-3p (*p* = 0.006; Figure 6E) and an 85.9% decrease in mature miR-495-3p (*p* = 0.003; Figure 6F). 

### 2.7. Scratch-Wound Healing in CIRBP Knockdown or lncRNA-CIRBP-AS1 Knockdown HUVECs after 4 Hours of siRNA Treatment

As knockdown of both *CIRBP* and its antisense had similar effects on microRNA expression and angiogenesis, as well as on each other, it cannot be concluded which of the two was the main effector. Therefore, the scratch-wound healing experiments described above were repeated using a much shorter transfection time of 4 h. We confirmed that a shorter transfection time would allow for direct effects on gene expression but not yet for indirect effects (Supplemental Materials Figure S1). 

**Figure 7:**
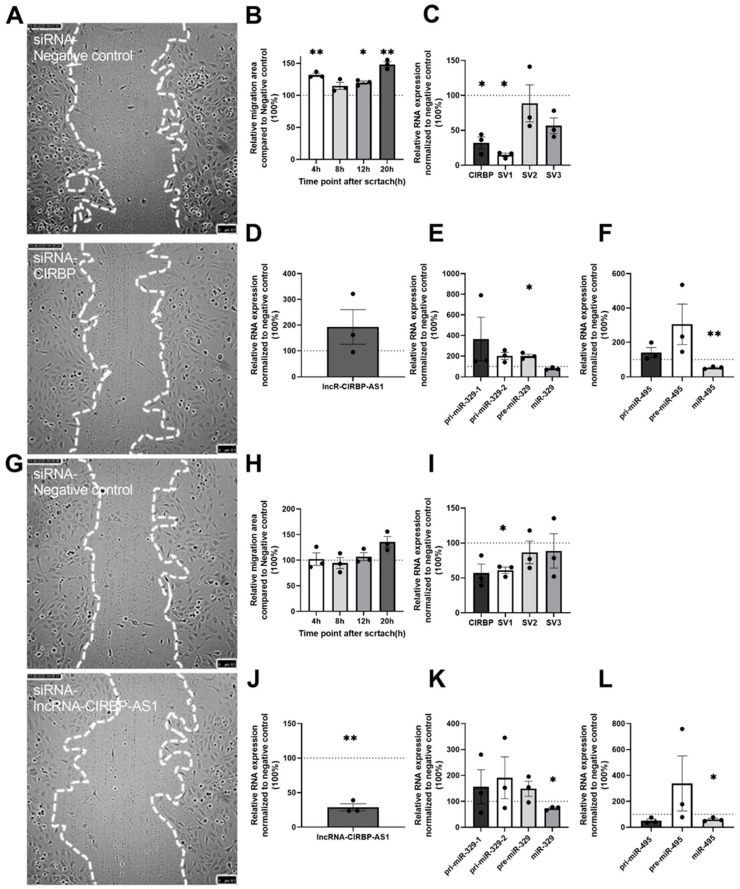
Four-hour transfection of siRNA on HUVECs: (**A**) Representative image of wound healing (*n* = 3) at 20 h after scratch on HUVECs, and (**B**) quantification of the migration area at different timepoints, treated over 4 h with siRNA targeted to *CIRBP* and the negative control. White, dotted lines mark the edge of the HUVECs’ layer. (**C**) Total *CIRBP* expression and its splice variant expressions after the scratch assay, normalized to GAPDH. (**D**–**F**) After the scratch assay, the pri-miRNA, pre-miRNA, and mature microRNA expression levels of miR-329 and miR-495 were measured and normalized to U6; lncRNA-*CIRBP*-AS1 were measured and normalized to GAPDH. (**G**) Representative image of wound healing (*n* = 3) at 20 h after scratch on HUVECs, and (**H**) quantification of the migration area at different timepoints, treated over 4 h with siRNA targeted to *CIRBP* and the negative control. White, dotted lines mark the edge of the HUVECs’ layer. (**I**) Total *CIRBP* expression and its splice variant=s after the scratch assay, normalized to GAPDH. (**J**–**L**) After the scratch assay, the pri-miRNA, pre-miRNA, and mature microRNA expression levels of miR-329 and miR-495 were measured and normalized to U6; lncRNA-*CIRBP*-AS1 were measured and normalized to GAPDH. Data show the percentage compared to siRNA and the negative control group. Data are presented as the mean ± SEM; * *p* < 0.05; ** *p* < 0.01; one-sample *t*-tests (two-tail).

*CIRBP* inhibition, again, enhanced HUVEC migration significantly (31.8%, *p* = 0.006 at 4 h; 14.7%, *p* = 0.11 at 8 h; 19.8%, *p* = 0.015 at 12 h; 48.2%, *p* = 0.007 at 20 h, Figure 7A,B). 

*CIRBP* and *CIRBP-SV1* expression were downregulated compared to the control siRNA (67.8% decrease, *p* = 0.015; 85.2% decrease, *p* < 0.001, respectively), and *CIRBP-SV3* showed a trend towards decreased expression (43.4%; *p* = 0.06) (Figure 7C). Expression of the lncRNA-*CIRBP*-AS1 was unaffected (Figure 7D); however, both precursors of the microRNAs, pre-miR-329 (101%; *p* = 0.03) and pre-miR-495, appeared upregulated, whereas the mature miR-495-3p was, again, downregulated (46.87%; *p* = 0.007) (Figure 7E,F), indicating reduced processing from precursor to mature microRNA in accordance with our previous study [25]. When HUVECs were transfected with an siRNA against lncRNA-*CIRBP*-AS1 for only 4 h, there was no significant difference in the cell migration area (Figure 7G,H). 

Expression of *CIRBP-SV1* and lncRNA-*CIRBP*-AS1 were downregulated by 39.0% (*p* = 0.014) and 71.1% (*p* = 0.005), respectively (Figure 7I,J), and the expression of both miR-329-3p and miR-495-3p also decreased by 26.7% (*p* = 0.02) and 39.9% (*p* = 0.045), respectively (Figure 7K,L).

However, the lack of an effect on cell migration of siRNA-lncRNA-*CIRBP*-AS1 after 4 h of transfection strongly supports the idea that *CIRBP*, and not lncRNA-*CIRBP*-AS1, was the main effector in *CIRBP*-mediated effects on angiogenesis.

## 3. Discussion

In this study, we confirmed the previous findings that deficiency in *CIRBP* leads to enhanced angiogenesis in a murine hindlimb ischemia model. We demonstrated that during ischemia, *CIRBP* contributed to the regulation of the angiomiRs, miR-329-3p and miR-495-3p, both in vivo and in vitro. Importantly, we demonstrated increased angiogenic activity upon *CIRBP* knockdown in human endothelial cells. Furthermore, we investigated the complex *CIRBP* gene sequence in humans more closely and looked in detail at the regulation of different *CIRBP* splice variants as well as a regulatory antisense lncRNA, lncRNA-*CIRBP*-AS1.

The hindlimb ischemia model is a classical method to investigate post-ischemic neovascularization, commonly employed as a model for PAD in humans [26]. Even though *Cirp^−/−^* mice display enhanced angiogenesis after induction of ischemia, faster blood flow recovery in *Cirp^−/−^* mice compared to wild-type C57/BL6 mice was not observed. Most likely, this can be attributed to the fact that during hindlimb ischemia and PAD, blood flow recovery depends more strongly on arteriogenesis than on angiogenesis [27]. Nonetheless, enhanced angiogenesis can still be highly beneficial for PAD patients, as the available blood is distributed better throughout the affected tissues and as enhanced angiogenesis can prevent non-healing wounds and ulcers, which are a common and serious complication in PAD patients [28]. Indeed, it was shown in *Cirp^−/−^* mice that the inflammatory response in tissue wound healing was faster than in wild-type mice. More CD31 expression, as a marker of endothelial cells and, thus, angiogenesis, was observed in the wounds of *Cirp^−/−^* mice [29], although the exact mechanism of action for *CIRBP* in wound healing was not elucidated. The pro-inflammatory function of *CIRBP* under stress conditions has already been reported by Qiang et al. [30]. Furthermore, *CIRBP* was reported to influence changes in leukocyte recruitment and macrophage polarization in direction to regenerative M2-like macrophages, thus regulating angiogenesis and tissue regeneration [5]. Moreover, the authors reported that *CIRBP* binds TLR4, MD2, and the TLR4/MD2 complex, which are known to stimulate neovascularization and can be found on both macrophages and neutrophils [31]. However, *CIRBP* acts through other angiogenesis-related pathways as well and, furthermore, the *CIRBP* gene in humans has a more complex structure than in mice, as it has several splice variants that result in different proteins as well as an antisense lncRNA. Their role has not been investigated in angiogenesis yet.

In previous studies, our group showed that *CIRBP* regulates the processing and expression of microRNAs, miR-329-3p and miR-495-3p [25], which both play an important role in angiogenesis [21]. We expected *CIRBP* to affect angiogenesis by regulating the expression of miR-329-3p and miR-495-3p. Importantly, we also reported enhanced macrophage attraction and influx into the vessel wall upon miR-495-3p inhibition [22], which could help explain the effects observed in the studies by Kübler et al. described above. In the current study, we showed that *CIRBP* was regulated under hypothermic conditions as was reported previously [7,9,10,11,12]. The novelty of this study, however, is that we showed this in human vascular endothelial cells that were subjected to hypothermic conditions, which frequently occurs in PAD patients [1]. The expression of the 14q32 microRNAs, miR-329-3p and miR-495-3p, did not increase under hypothermia in HUVECs, however, even though the expression of their reported post-transcriptional regulator *CIRBP* increased. In contrast, silencing of *CIRBP*, using an siRNA, did lead to a decrease in miR-495-3p expression. Likely, the processing rate of 14q32 microRNAs was already at an optimum level under normothermic conditions, which can explain that an increase in *CIRBP* did not induce more processing of precursor microRNAs. The dramatic decrease in *CIRBP* expression following siRNA treatment, on the other hand, did result in insufficient processing of miR-495-3p. Surprisingly, expression of miR-329-3p was still unaffected in vitro in human cells, even though we did observe a trend towards accumulation of the primary microRNA, pri-miR-329-1, which would correspond with the inhibition of microRNA biogenesis. In vivo in mice, on the other hand, we could clearly see that ischemia-induced upregulation of both miR-329-3p and miR-495-3p was blocked quite efficiently in *Cirp^−/−^* mice.

When we speculate what these findings could mean for human PAD, *CIRBP* expression would likely be increased, as patients suffer from cold extremities [1]. We found that hypothermia caused *CIRBP* expression upregulation. Such an increase in *CIRBP*, likely accompanied by continuously high miR-329-3p and possible miR-495-3p expression, would inhibit efficient angiogenesis and wound healing in PAD patients, with, for example, non-healing ulcers. Meanwhile, others have reported that warm temperatures (42 °C) can decrease *CIRBP* expression in male germ cells [32]. Therefore, one could imagine that hyperthermia leads to decreased *CIRBP* expression in the leg, followed by subsequent increases in angiogenesis. Thus far, heat therapy was shown to be of potential benefit to PAD patients by enhancing leg blood flow and improving muscle function [33,34]. 

We further assessed the potential pro-angiogenic features of *CIRBP* knockdown in human primary endothelial cells. Indeed, cell-migration assays as well as tube-formation assays in HUVECs showed an increase in angiogenic potential following *CIRBP* downregulation. These findings are in line with those found in vivo. Looking at human cells, however, also allowed us to look into changes in the *CIRBP* splice variants (*CIRBP-SVs*) and its antisense long noncoding RNA (lncRNA-*CIRBP*-AS1). *CIRBP* has four splice variants that alter the coding sequence. Of these four variants, *CIRBP-SV1* specifically showed a trend towards upregulation after 24 and 48 h of hypothermia. When we knocked down *CIRBP* using an siRNA, we again observed specific knockdown of *CIRBP-SV1*. This was unexpected, as the binding site of the siRNA was predicted to target an mRNA sequence that is present in all four splice variants. Although we cannot explain the siRNA’s preference for *CIRBP-SV1*, we can conclude that the majority of *CIRBP*’s effects on angiogenesis were elicited through *CIRBP-SV1*.

The antisense strand of the human *CIRBP* gene also encodes a long noncoding RNA, lncRNA-*CIRBP*-AS1, the function of which has not yet been elucidated. We observed a trend towards upregulation of lncRNA-*CIRBP*-AS1 under hypothermic conditions, although the response to hypothermia was slower than that of *CIRBP* itself. After *CIRBP* knockdown, lncRNA-*CIRBP*-AS1 expression decreased, and after lncRNA-*CIRBP*-AS1 knockdown, *CIRBP* expression decreased; inhibition of lncRNA-*CIRBP*-AS1 with an siRNA resulted in simultaneous downregulation of total *CIRBP* and of *CIRBP-SV1*, and *CIRBP-SV3* in particular. Furthermore, lncRNA-*CIRBP*-AS1 knockdown resulted in a decreased expression of both mature miR-329-3p and miR-495-3p. More importantly, silencing of lncRNA-*CIRBP*-AS1, like *CIRBP* itself, resulted in increased angiogenesis in HUVECs. 

Likely, a positive feedback loop supports the transcription of the *CIRBP* locus, where *CIRBP* and lncRNA-*CIRBP*-AS1 induce each other’s expression. In order to elucidate which of the two is the main effector in the enhanced angiogenic potential of HUVECs, we separated their mutual effects by shortening the siRNA transfection duration, and we found that *CIRBP* knockdown still had the same effect on angiogenesis and regulation of microRNA expression, while lncRNA-*CIRBP*-AS1 knockdown could no longer improve angiogenesis, even though miR-329-3p expression was already reduced significantly. Combining these findings, we conclude that the effects of lncRNA-*CIRBP*-AS1 downregulation on angiogenesis were most likely indirect. We can also conclude, however, that lncRNA-*CIRBP*-AS1 can directly impact *CIRBP* expression and, likely, also miR-329-3p expression. The mechanisms behind this regulation remain to be determined. 

In conclusion, our findings confirm the previously reported increase in post-ischemic angiogenesis in *Cirp^−/−^* mice. We showed that *CIRBP* directly regulated the angiomiRs microRNAs, miR-329-3p and miR-495-3p, in vivo. In addition, we validated these findings in human primary endothelial cells for the first time. Furthermore, we showed that *CIRBP-SV1* was the splice variant that predominantly regulates *CIRBP*’s effects on both microRNA expression and angiogenesis. Finally, the lncRNA-*CIRBP*-AS1 can also impact angiogenesis, but these effects are likely caused by directing changes in *CIRBP-SV1* and, subsequently, miR-329-3p and miR-495-3p. 

## 4. Materials and Methods

### 4.1. Animal Experiments

All animal experiments were approved by the Committee on Animal Welfare of the Leiden University Medical Center (Leiden, The Netherlands) and were performed in accordance with the Directive 2010/63/EU of the European Parliament and Dutch government guidelines. *Cirp^−/−^* embryos on a C57BL6/J background were kindly provided by Jun Fujita’s lab (Kyoto University, Kyoto, Japan) [35]. WT C57BL/6 mice (*n* = 10, male, aged 8–10 weeks) and *Cirp^−/−^* mice (*n* = 11, male, aged 8–10 weeks) were bred in the LUMC’s in-house breeding facility and had free access to water and regular chow. 

### 4.2. HLI Model

Before surgery, mice were anesthetized via an intraperitoneal injection of midazolam (5 mg/kg; Roche Diagnostics, Almere, The Netherlands), medetomidine (0.5 mg/kg; Orion, Espoo, Finland), and fentanyl (0.05 mg/kg; Janssen Pharmaceuticals, Beerse, Belgium). Unilateral HLI was induced by double ligation of the left femoral artery, proximal to the superficial epigastric artery and proximal to the bifurcation of the popliteal and saphenous artery. After surgery, mice were given a subcutaneous injection of flumazenil (0.5 mg/kg, Fresenius Kabi, Utrecht, The Netherlands) and atipamezol (2.5 mg/kg, Orion) to antagonize anesthesia. Buprenorphine (0.1 mg/kg, MSD Animal Health, Boxmeer, The Netherlands) was given after surgery for pain relief [36].

### 4.3. Laser Doppler Perfusion Measurements

Blood flow recovery to the paw was measured over time using laser Doppler perfusion imaging (LDPI) (Moor Instruments, Axminster, United Kingdom) at day 0 (before and after ligation), 3, 7, 10, 14, 21, and 28. Before measurements, mice were anesthetized with an intraperitoneal injection of midazolam (5 mg/kg, Roche Diagnostics) and medetomidine (0.5 mg/kg, Orion). Mice were placed in a double-glassed pot that was perfused with water at 37 °C for 5 min prior to each measurement. LDPI measurements in the ligated paw were normalized to measurements of the unligated paw as an internal control. After LDPI, anesthesia was antagonized by subcutaneous injection of flumazenil (0.5 mg/kg) and atipamezole (2.5 mg/kg).

At day 28, after the last LDPI measurement, mice were injected with fentanyl (0.05 mg/kg) and sacrificed via retro-orbital bleeding. The proximal half of the adductor muscle was harvested and fixed in 4% formaldehyde. The distal half of the adductor muscle and the soleus muscle were harvested and snap-frozen on dry-ice. 

### 4.4. Immunohistochemical Staining

Adductor muscles were embedded in paraffin, and 5 μm thick sections were cut for histological analysis. Smooth muscle cells were stained with primary antibody mouse anti-mouse α-SMA (Dako, 1:1000). Rabbit anti-mouse HRP (Dako, 1:300) was used as the secondary antibody. Slides were scanned with the Pannoramic MIDI digital slide scanner (3DHistech). The number and lumen diameter of α-SMA positive vessels were analyzed by Pannoramic viewer software (3DHistech, version: 2.3) with 20× magnification (3 sections per limb per mouse). The smallest diameter of vessel was measured in the picture as described previously [37].

Six μm-thick frozen soleus sections (3 sections per limb per mouse) were fixed in ice-cold acetone and stained using primary antibody anti-CD31 biotin (Biolegend, 102503, 1:100) and an avidin–biotin complex (ABC) kit (Vector, Burlingame, CA, USA). Slides were scanned with the Pannoramic MIDI digital slide scanner (3DHistech). Random snapshots (3 per section) were taken by the Pannoramic viewer software (3DHistech) with 40× magnification (6–9 images per limb per mouse). The CD31 positive area was quantified by ImageJ as described previously [38].

### 4.5. Isolation of Venous Endothelial Cells (HUVECs)

Primary human vascular cells were isolated as described earlier by Welten et al. [21]. In brief, for HUVEC isolation, the vein was inserted with a cannula and flushed with sterile PBS. The vessel was infused with 0.075% collagenase type II (Worthington) and incubated at 37 °C for 20 min. The collagenase solution was collected, and the vessel was flushed with PBS in order to collect all detached endothelial cells. The cell suspension was centrifuged at 400× *g* for 5 min, and the pellet was resuspended in HUVEC culture medium (EBM-2 Basal Medium (CC-3156) and EGMTM-2 SingleQuots^TM^ Supplements (CC-4176), Lonza, Walkersville, MD, USA). HUVECs were cultured in plates coated with 1% fibronectin from bovine plasma (Sigma, Amsterdam, The Netherlands).

### 4.6. Primary HUVEC Cell Culture

HUVECs were cultured at 37 °C in a humidified 5% CO_2_ environment with HUVEC culture medium (EBM-2 Basal Medium (CC-3156) and EGMTM-2 SingleQuots^TM^ Supplements (CC-4176), Lonza). Media were refreshed every 2–3 days. Cells were passed using trypsin (Sigma) at 70–80% confluency. HUVECs were used for the scratch-wound healing assay at passage three. HUVECs were stored up to passage three in 90% heat inactivated New Born Calf Serum (NBSCi) (Sigma) and 10% DMSO (Sigma).

### 4.7. Hypothermic HUVEC Cell Culture

Primary HUVECs were seeded in 12-well plates coated with 1% fibronectin at 100,000 cells per well in culture medium. After overnight incubation at 37 °C, cells were washed with PBS and new media were applied before putting the plates in the right incubator: the normothermic incubator at 37 °C and the hypothermic incubator at 32 °C, both humidified at 5% CO_2_ and 20% O_2_. After 24 or 48 h, cells were washed with PBS, and 0.5 mL TRIzol/well was added for RNA isolation. Each single condition was performed in triplicate, and the hypothermia experiment was performed three independent times.

### 4.8. CIRBP and lncRNA-CIRBP-AS1 Knockdown with siRNA Transfection In Vitro

Primary HUVECs were seeded in 12-well plates coated with 1% fibronectin at 100,000 cells per well in culture medium. After 24 h, cells were washed with PBS, and each well was incubated with 900 µL Opti-MEM medium with 10% NBSCi and 1% penicillin/streptomycin and, after 10 min of incubation, 100 µL of transfection medium (94 µL Opti-MEM with 3 µL of Lipofectamine RNAiMax (Life Technologies, Bleiswijk, The Netherlands) and 3 µL of siRNA) was added. The final siRNA concentration per well was 30 nM. The siRNAs used were siRNA-*CIRBP* (sasi-172352), siRNA-lncRNA-*CIRBP*-AS1 (sasi-208901), and siRNA negative control (Mission Universal Negative Control #1) (all Sigma–Aldrich, Amsterdam, The Netherlands). After addition of transfection agents, cells were put in the incubator at 37 °C for the required time.

### 4.9. Migration Assay–Scratch Wound Healing 

Primary HUVECs were seeded in 12-well plates coated with 1% fibronectin at 150,000 cells per well in HUVEC culture medium. After 24 h, the media were replaced with transfection medium as previously described. Then, transfection was conducted, and a scratch wound was performed across the diameter of each well using a p200 pipette tip. Next, cells were washed with PBS and fresh starving medium, and EBM-2 (Lonza) containing only 0.2% FBS and 1% gentamicin amphotericin of the provided BulletKit was added. In order to monitor scratch-wound closure, live phase-contrast microscopy (Axiovert 40C, Carl Zeiss, Oberkohen, Germany) was used for taking pictures at 0 and 18 h after introducing the scratch wound. In addition, a live cell microscope (Leica AF6000, Leica Microsystems, Tokyo, Japan) was used for taking picture every 4 h after scratch until 20 h in the timeline experiment. Pictures were taken in the same location at two positions in each well. Where necessary, pictures were contrast-enhanced using Microsoft PowerPoint. Scratch size was calculated using the wound healing tool macro for ImageJ. Finally, cells were washed with PBS, and 0.5 mL TRIzol/well was added for RNA isolation. Each single scratch assay condition was performed in triplicate, and the scratch-wound healing assay was performed three independent times. 

### 4.10. Tube-Formation Assay

Tube-formation assay was performed using HUVECs at passage three. At confluence, cells were transfected as described above with Lipofectamine RNAiMax and siRNA-*CIRBP*, lncRNA-*CIRBP*-AS1, or siRNA negative control. After 24 h, cells were counted and seeded on solidified Geltrex^TM^ (ref: A14132-02, Gibco) in a 96-well plate at 15,000 cells per well. Photos were taken using live phase-contrast microscopy at 12 h after seeding and quantified using the ImageJ Angiogenesis Analyzer. Each single tube-formation assay was performed in 6 wells per condition, and the tube-formation assay was performed three independent times.

### 4.11. Aorta Ring Assay

Thoracic aortas were isolated from *Cirp^−/−^* and wild-type mice, aged 4–5 weeks, after exsanguination under anesthesia via an intraperitoneal injection of midazolam (5 mg/kg; Roche Diagnostics), medetomidine (0.5 mg/kg; Orion), and fentanyl (0.05 mg/kg; Janssen Pharmaceuticals). Vessels were washed with Opti-MEM medium containing 1% penicillin/streptomycin and were cut transversely. Consequently, aorta rings were obtained 0.5–1 mm in width and incubated in Opti-MEM medium with 1% penicillin/streptomycin overnight at 37 °C. Collagen (type I, Merck Millipore, Darmstadt, Germany) was diluted to a concentration of 1 mg/mL with DMEM and 1% penicillin/streptomycin, and the pH was adjusted with 5 N NaOH to 7. Aortic rings were placed in 96-well plates coated with 75 μL collagen matrix as described previously [39]. After 1 h, 150 μL Opti-MEM supplemented with 2.5% FBS (PAA Laboratories, Pasching, Austria), penicillin–streptomycin (PAA Laboratories), and with or without vascular endothelial growth factor (in-house production and purification) of 10 or 30 ng/mL were added to the designated wells. Media were refreshed on days 3 and 5. Microvessel outgrowth was quantified after 7 days on photographs taken by live phase-contrast microscopy (Axiovert 40C, Carl Zeiss). The counting of microvessels started from a specific point on the ring, and each microvessel emerging from the ring was counted as a sprout, and individual branches arising from each microvessel counted as a separate sprout, working around the ring clockwise.

### 4.12. RNA Isolation

RNA isolation of cultured cells or tissue was performed by standard TRIzol–chloroform extraction according to the manufacturer’s instructions (Thermo Fisher Scientific, Wilmington, DE, USA). RNA concentrations were measured using the Nanodrop^TM^ 1000 Spectrophotometer (Thermo Fisher Scientific). 

### 4.13. MicroRNA Quantification

For microRNA quantification of miR-329-3p and miR-495-3p, RNA was reversed transcribed using the Taqman^TM^ MicroRNA Reverse-Transcription Kit (Thermo Fisher Scientific) and, subsequently, quantified using microRNA-specific Taqman^TM^ qPCR kits (Thermo Fisher Scientific) on the VIIa7 (Thermo Fisher Scientific). MicroRNA expression was normalized against U6 small nuclear RNA. 

### 4.14. mRNA, pri-microRNA, and pre-microRNA Quantification

For quantification of the expression levels of *CIRBP*, *CIRBP-SVs*, lncRNA-*CIRBP*-AS1, primary microRNAs (pri-miRs), and pre-miRs, RNA was reverse transcribed using a “High-Capacity RNA to cDNA Kit” (Thermo Fisher Scientific) and quantified by qPCR using SybrGreen reagents (Qiagen, Hilden, Germany) on the VIIa7. *CIRBP*, *CIRBP-SVs*, *PCNA*, and lncRNA-*CIRBP*-AS1 expressions were normalized against GAPDH; pri-miRs and pre-miRs expressions were normalized to U6. Primer sequences are provided in Table 1.

### 4.15. Statistical Analyses

Data are presented as the mean ± SEM. Indicated differences had the following levels of significance: * *p* <0.05; ** *p* < 0.01; *** *p* < 0.001. All tests were performed with a significance level of α = 0.05.

One-sample *t*-tests were performed to test differences between treated groups that were expressed relative to the negative control treatment, which was set to 100%. One-sample *t*-tests (two tail) were used in the knockdown experiments, scratch assay, and tube-formation assay. One-sample *t*-tests (one tail) were used in the hypothermia experiment. 

Differences in scratch wound healing and PCNA levels between groups were assessed using independent sample Student’s *t*-tests.

Two-way ANOVA tests were performed to detect statistically significant differences among multiple groups. These tests were used to compare miRNA-329-3p and -495-3p expression levels of the soleus of mice subjected to HLI surgery at different timepoints.

## Figures and Tables

**Table 1:** Sequences of primers used for qPCR and the siRNAs used for knockdown.

Primers	Forward Sequence	Reverse Sequence
HSA-CIRBP	TTGACACCAATGAGCAGTCG	GGCATCCTTAGCGTCGTCAA
HSA-splice variant 1	CGTGGGTTCTCTAGAGGAGGA	CTCGTTGTGTGTAGCGTAACTG
HSA-splice variant 2	CGTGGGTTCTCTAGAGGAGGA	CGCCCTCGGAGTGTGACTTA
HSA-splice variant 3	CGTGGGTTCTCTAGAGGAGGA	TCAACCGTAACTGTCATAACTG
HSA-splice variant 4	GTAGACCAGGCAGGAGGAG	CGCCCTCGGAGTGTGACTTA
HSA-lncRNA-CIRBP-AS1	CAATGGGAAAAGGAGGAAACT	CCTTGTAAAGCTGGTTCTCCA
GAPDH	CACCACCATGGAGAAGGC	AGCAGTTGGTGGTGCAGGA
HSA-pri-miR-329-1	TGGGGAAGAATCAGTGGTGT	GACCAGAAGGCCTCCAAGAT
HSA-pri-miR-329-2	TGTCAAGTTTGGGGAAGGAA	GACCAGAAGGCCTCCAAGAT
HSA-pre-miR-329	TGAAGAGAGGTTTTCTGGGTTT	ACCAGGTGTGTTTCGTCCTC
HSA-pri-miR-495	CTGACCCTCAGTGTCCCTTC	ATGGAGGCACTTCAAGGAGA
HSA-pre-miR-495	GCCCATGTTATTTTCGCTTT	CCGAAAAAGAAGTGCACCAT
U6	AGAAGATTAGCATGGCCCCT	ATTTGCGTGTCATCCTTGCG
siRNA CIRPB	GAGUCAGAGUGGUGGCUAC	
siRNA lncRNA-CIRBP-AS1	CAGGACCCUCACUCACUA	

## Data Availability

All data are included in the manuscript or available upon request.

## Supplementary Materials

The following are available online at https://www.mdpi.com/article/10.3390/ijms222312678/s1.
