# Supplementary material for: Cold-Inducible RNA-Binding Protein but Not Its Antisense lncRNA Is a Direct Negative Regulator of Angiogenesis In Vitro and In Vivo via Regulation of the 14q32 angiomiRs—microRNA-329-3p and microRNA-495-3p
Source: Int J Mol Sci. 2021 Nov 24;22(23):12678. doi: 10.3390/ijms222312678 (PMC8657689; doi:10.3390/ijms222312678)
Supplement: Supplementary file 1 [file ijms-22-12678-s001.zip › ijms-1418041-supplementary.pdf]

Supplemental Figure S1

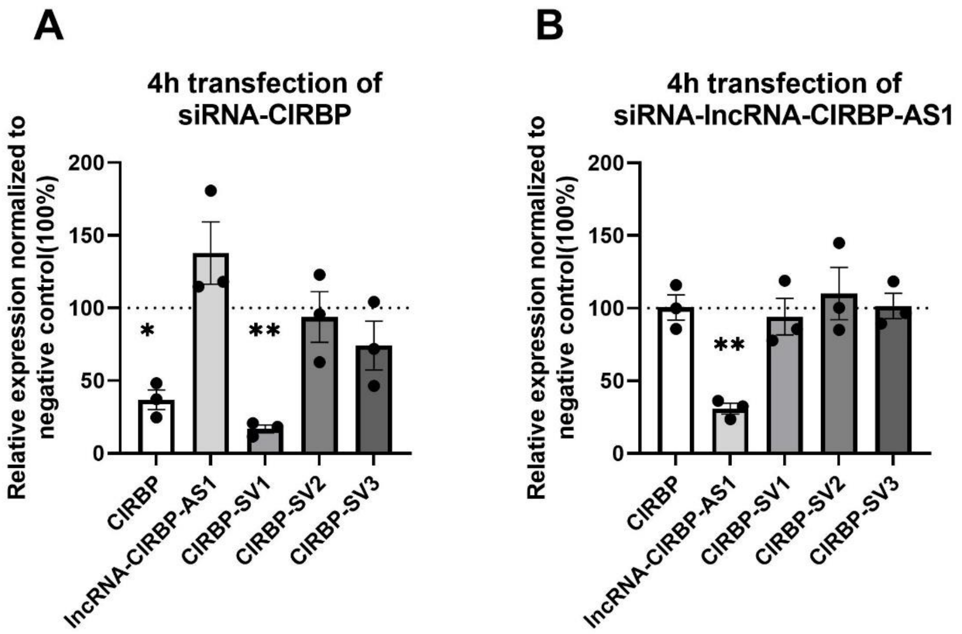

Figure S1. siRNA transfection for 4 hours in HUVECs. (A) Relative expression of total CIRBP, its splice variants, and lncRNA-CIRBP-AS1 after siRNA-CIRBP transfection, normalized to GAPDH. (B) Relative expression of total CIRBP, its splice variants, and lncRNA-CIRBP-AS1 after siRNA-lncRNA-CIRBP-AS1 transfection, normalized to GAPDH. Data show the percentage compared to the siRNA negative control group. Data are presented as mean ± SEM, *$p < 0.05$, **$p<0.01$, by one-sample t-tests (two-tail).
